# *Hippophae rhamnoides* L. Fruits Reduce the Oxidative Stress in Human Blood Platelets and Plasma

**DOI:** 10.1155/2016/4692486

**Published:** 2016-01-10

**Authors:** Beata Olas, Bogdan Kontek, Paulina Malinowska, Jerzy Żuchowski, Anna Stochmal

**Affiliations:** ^1^Department of General Biochemistry, Faculty of Biology and Environmental Protection, University of Łódź, 90-236 Łódź, Poland; ^2^Department of Biochemistry, Institute of Soil Science and Plant Cultivation, State Research Institute, 24-100 Puławy, Poland

## Abstract

Effects of the phenolic fraction from* Hippophae rhamnoides* fruits on the production of thiobarbituric acid reactive substances (TBARS, a marker of lipid peroxidation) and the generation of superoxide anion (O_2_^−∙^) in human blood platelets (resting platelets and platelets stimulated by a strong physiological agonist, thrombin) were studied* in vitro*. We also examined antioxidant properties of this fraction against human plasma lipid peroxidation and protein carbonylation induced by a strong biological oxidant, hydrogen peroxide (H_2_O_2_) or H_2_O_2_/Fe (a donor of hydroxyl radicals). The tested fraction of* H. rhamnoides* (0.5– 50 *µ*g/mL; the incubation time: 15 and 60 min) inhibited lipid peroxidation induced by H_2_O_2_ or H_2_O_2_/Fe. The* H. rhamnoides* phenolic fraction inhibited not only plasma lipid peroxidation, but also plasma protein carbonylation stimulated by H_2_O_2_ or H_2_O_2_/Fe. Moreover, the level of O_2_^−∙^ in platelets significantly decreased. In comparative experiments, the* H. rhamnoides* fraction was a more effective antioxidant than aronia extract or grape seed extract (at the highest tested concentration, 50 *µ*g/mL). The obtained results suggest that* H. rhamnoides* fruits may be a new, promising source of natural compounds with antioxidant and antiplatelet activity beneficial not only for healthy people, but also for those with oxidative stress-associated diseases.

## 1. Introduction

*Hippophae rhamnoides* L. (sea buckthorn), naturally occurring throughout Asia and Europe, is a member of the Elaeagnaceae family [1]. Due to its multiple biological properties (anticancer, antimicrobial, and cardioprotective) [1–6],* H. rhamnoides* has been used in traditional medicine for a long time [1, 7, 8]. This plant has been used extensively in traditional oriental system of medicine for treatment of asthma, skin diseases, lung disorders, and gastric ulcers. All parts of* H. rhamnoides*, including fruits, are considered to be a rich source of different bioactive compounds [5, 6, 9]. It contains phenolic compounds, lipids, and vitamin C; phenolics are responsible for its pharmacological activity [1, 5, 8, 10]. Moreover, dietary intake of phenolic compounds may be viewed as an important strategy for treating pathological conditions or delaying their development, as well as a chance for disease prevention in many paradigms. Experiments of Eccleston et al. [11] reported that the fruit juice of* H. rhamnoides *had a protective effect against cardiovascular diseases, but the mechanism is not fully clear as yet. It is very important that* H. rhamnoides* is one of the most representative economy crops for its wide uses of biological diversity and abundance of resource [12].

In this study, we focused on fruits of* H. rhamnoides*, which might be of great interest. The objective was to investigate the antioxidant activity of the phenolic fraction from* H. rhamnoides* fruits against the effect of a strong biological oxidant, hydrogen peroxide (H_2_O_2_) or H_2_O_2_/Fe (the donor of hydroxyl radicals), on human plasma lipids and proteins. Moreover, we determined the effect of the phenolic extract on a nonenzymatic lipid peroxidation in resting blood platelets and enzymatic lipid peroxidation, arachidonic acid metabolism (measured by the level of thiobarbituric acid reactive substances (TBARS)), in blood platelets activated by thrombin (as a strong physiological agonist). We also investigated the* in vitro* effect of the tested fraction on the superoxide anion (O_2_^−∙^) production in resting blood platelets and platelets activated by thrombin. Experimental models used in this study were similar to reactions which take place in human plasma and blood platelets under oxidative stress conditions or during a blood platelet activation. Moreover, blood platelets and their activation are important in hemostasis, and in various diseases, that is, cardiovascular diseases. The action of the fraction from* H. rhamnoides* fruits was also compared to activities of two phenolic extracts: a commercial extract from the berries of* Aronia melanocarpa* (Aronox) and a grape seed extract with antioxidative and antiplatelet properties [13–15]. The range of tested concentrations of the* H. rhamnoides* fraction (0.5–50 *µ*g/mL) or the other two extracts (50 *µ*g/mL) can be achieved in plasma by way of supplementation with phenolic compounds.

## 2. Material and Methods

### 2.1. Chemicals

Cytochrome c, dimethylsulfoxide (DMSO), thiobarbituric acid (TBA), H_2_O_2_, and formic acid (LC-MS grade) were purchased from Sigma (St. Louis, MO., USA). Methanol (isocratic grade) and acetonitrile (LC-MS grade) were acquired from Merck (Darmstadt, Germany). All other reagents represented analytical grade and were provided by commercial suppliers.

A stock solution of* A. melanocarpa* extract (commercial product, Aronox, by Agropharm Ltd., Poland; batch number 020/2007k) was made in H_2_O at a concentration of 5 mg/mL, kept frozen, and then used for experiments. Total content of phenolics in the phenolic-rich powder used in this study amounted to 309.6 mg/g of extract, including phenolic acids (isomers of chlorogenic acid), 149.2 mg/g of extract, anthocyanins (anthocyanin glycosides: cyanidin 3-galactoside, cyanidin 3-glucoside, cyanidin 3-arabinoside, and cyanidin 3-xyloside), 110.7 mg/g, and flavonoids (quercetin glycosides), 49.7 mg/g of extract. The HPLC determination of the phenolic-rich extract from berries of* A. melanocarpa* was described previously [13–15].

The grape seed extract was supplied by Bionorica (Germany) with a total content of phenolics equalling 500 mg/g of extract [15]. A stock solution of grape seed extract was prepared in 50% DMSO.

### 2.2. Plant Material

Sea buckthorn (*Hippophae rhamnoides* L.) berries were obtained from a horticultural farm in Sokółka, Podlaskie Voivodeship, Poland (53°24′N, 23°30′E). Fruits were freeze-dried and stored in a refrigerator.

### 2.3. Preparation of the Fraction of Phenolic Compounds

Freeze-dried sea buckthorn fruits (800 g) were subjected to cold extraction with 4 L of 80% methanol (v/v; 24 h), assisted with ultrasonic treatment (2 × 10 min). The plant material was further extracted with boiling 80% methanol (v/v; 4 L; 1 h), under reflux. Both extracts were combined, filtered, and concentrated in a rotary evaporator (Heidolph, Schwabach, Germany) to remove the organic solvent. The residue was applied onto a short LiChroprep 40−63 *μ*m RP-18 column (Millipore Corp., Bedford, MA) and equilibrated with water. The column was washed with water to remove highly polar extract constituents, and bound phenolic compounds were eluted with 50% methanol (v/v). The obtained 50% methanol eluate was concentrated by a rotary evaporation and subsequently freeze-dried, to yield 10.19 g of dry phenolic fraction.

### 2.4. LC-MS Analysis

The composition of the phenolic fraction of sea buckthorn fruits was determined using an HPLC-ESI-MS/MS method. Chromatographic analyses were carried out with the application of a Thermo Finnigan Surveyor HPLC system, equipped with a PDA detector and coupled with a Thermo LCQ Advantage Max ion-trap mass spectrometer. Separation was performed on a Waters XBridge BEH C18 column (3.0 × 150 mm, 2.5 *µ*m), maintained at 50°C. The injection volume was 5 *µ*L. The elution (300 *μ*L min^−1^) was carried out with a gradient of solvent B (acetonitrile with 0.1% FA) in solvent A (MilliQ water with 0.1% FA): 0–5 min, 5% B; 5–85 min, 5–60% B; 85–95 min, 60% B. The UV-Vis spectra were recorded from 200 to 600 nm. Mass spectral analyses were performed in the negative ionization mode with the following parameters: capillary temperature 260°C, spray voltage 3.9 kV, capillary voltage −47 V, tube lens offset −60 V, sheath gas flow 70 (arbitrary units), and sweep gas flow 10 (arbitrary units). The MS scan covered a range from* m/z* 150 to* m/z* 2000; the MS/MS spectra were acquired in the Data-Dependent Analysis (DDA) mode. Flavonoids were identified on the basis of their MS and UV spectra, as well as literature data [16, 17].

Quantitative analyses were carried out using UHPLC-UV-MS method. Chromatographic separations were performed on an ACQUITY UPLC chromatograph, consisting of a binary solvent manager, autosampler, column manager, and photodiode array detector (Waters, Milford, MA, USA), and coupled with a triple quadrupole mass detector (ACQUITY TQD, Waters). Samples were separated on an ACQUITY HSS C18 (100 × 2.1 mm, 1.8 *μ*m; Waters) column and maintained at 40°C. The injection volume was 2.5 *µ*L. The elution (400 *μ*L min^−1^) was carried out with a gradient of solvent B (acetonitrile with 0.1% FA) in solvent A (MilliQ water with 0.1% FA): 0–0.50 min, 1% B; 0.50–17.95 min, 1–35.5% B; 17.95–18.00 min, 35.5–99% B; 18.00–20.00 min, 99% B. Capillary voltage amounted to 3.0 kV, cone voltage 40 V, source temperature 140°C, desolvation temperature 350°C, cone gas flow (nitrogen) 100 L h^−1^, and desolvation gas flow 800 L h^−1^. PDA detection was used for the quantitation of phenolic compounds (*λ* = 350 nm for flavonoids, 254 nm for other phenolics). Since isorhamnetin derivatives are the main flavonoids found in sea buckthorn fruits, the standard curve of isorhamnetin 3-*O*-*β*-glucosyl(1→2)-*β*-galactoside was applied to calculate relative concentrations of individual flavonoids and the total content of other phenolic compounds. Analyses were performed in triplicate.

A stock solution of the investigated plant fraction was made in 50% DMSO. The final concentration of DMSO in samples was lower than 0.05% and its effects were determined in all experiments.

### 2.5. Blood Platelet and Plasma Isolation

Fresh human plasma was obtained from regular, medication-free donors at a blood bank (Łódź, Poland). Peripheral blood was also obtained from nonsmoking men and women (collected into ACD solution (citric acid/citrate/dextrose; 5 : 1; v/v; blood/ACD)). They had not taken any medications or addictive substances (including tobacco, alcohol, antioxidant supplementation, aspirin, or any other antiplatelet drugs). The protocol was approved by the Committee for Research on Human Subjects of the University of Łódź number 2/KBBN-UŁ/III/2014. Platelet-rich plasma (PRP) was prepared by centrifugation of fresh human blood at 250 ×g for 10 min at room temperature. Platelets were then sedimented by centrifugation at 500 ×g for 10 min at room temperature. The platelet pellet was washed twice with Tyrode's buffer (10 mM HEPES, 140 mM NaCl, 3 mM KCl, 0.5 mM MgCl_2_, 5 mM NaHCO_3_, 10 mM glucose, pH 7.4); afterwards, the platelets were suspended in the same buffer. The concentration of platelets in suspensions, estimated spectrophotometrically [18], amounted to 5 × 10^8^/mL.

Suspensions of blood platelets or plasma were incubated (15 or 60 min, at 37°C) with

- *H. rhamnoides* fraction at the final concentrations of 0.5–50 *μ*g/mL,
- *H. rhamnoides* fraction at the final concentrations of 0.5–50 *μ*g/mL plus 2 mM H_2_O_2_,
- *H. rhamnoides* fraction at the final concentrations of 0.5–50 *μ*g/mL plus 4.7 mM H_2_O_2_/3.8 mM Fe_2_SO_4_/2.5 mM EDTA,
- *H. rhamnoides* fraction at the final concentrations of 0.5–50 *μ*g/mL plus 5 U/mL thrombin (5 min, at 37°C),
- *Aronia* extract or grape seed extract at the final concentration of 50 *μ*g/mL,
- *Aronia* extract or grape seed extract at the final concentration of 50 *μ*g/mL plus 2 mM H_2_O_2_,
- *Aronia* extract or grape seed extract at the final concentration of 50 *μ*g/mL plus 4.7 mM H_2_O_2_/3.8 mM Fe_2_SO_4_/2.5 mM EDTA,
- *Aronia* extract or grape seed extract at the final concentration of 50 *μ*g/mL plus 5 U/mL thrombin (5 min, at 37°C).

### 2.6. Lipid Peroxidation Measurement

Lipid peroxidation was quantified by measuring the concentration of TBARS. Incubation of plasma or blood platelets (control, plant extract and H_2_O_2_- or H_2_O_2_/Fe-treated plasma, plant extract, and thrombin-treated platelets) was stopped by cooling the samples in an ice bath. Samples of plasma or platelets were transferred to an equal volume of cold 20% (v/v) trichloroacetic acid in 0.6 M HCl and centrifuged at 1200 ×g for 15 min. One volume of clear supernatant was mixed with 0.2 volume of 0.12 M thiobarbituric acid in 0.26 M Tris (pH 7.0), immersed in a boiling water bath for 15 min, and then absorbance was measured at 532 nm (Spectrophotometer UV/Vis Helios alpha Unicam) [19, 20]. The TBARS concentration was calculated using the molar extinction coefficient (*ε* = 156,000 M^−1^ cm^−1^).

### 2.7. Superoxide Anion Measurement

Cytochrome c reduction was used to measure O_2_^−∙^ generation in control and in platelets incubated with the tested compounds, as described earlier [21, 22]. Briefly, an equal volume of modified Tyrode's buffer, containing cytochrome c (160 *μ*M), was added to the platelet suspension. After incubation, the platelets were sedimented by centrifugation at 2000 ×g for 5 min and the supernatants were transferred to cuvettes. Cytochrome c reduction was measured spectrophotometrically at 595 nm. To calculate the molar concentration of O_2_^−∙^, the molar extinction coefficient for cytochrome c of 18,700 M^−1^ cm^−1^ was used.

### 2.8. Carbonyl Groups Measurement

Detection of carbonyl groups in plasma proteins was carried out according to the procedure of Dalle-Donne et al. [23].

### 2.9. Data Analysis

Statistical analysis was done using several tests. In order to eliminate uncertain data, the *Q*-Dixon test was performed. All the values in this study were expressed as mean ± standard error (SE). Statistical analysis was performed with one-way ANOVA for repeated measurements.

## 3. Results

The LC-MS analyses demonstrated that flavonoids were the dominant compounds in the phenolic fraction of sea buckthorn fruits and their total amount equalled 214.04 mg/g (Table 1). Among different flavonol glycosides, both isorhamnetin 3-*O*-hexoside-deoxyhexoside and isorhamnetin 3-*O*-hexoside were present in the highest amounts. Other phenolic compounds, including putative proanthocyanidins, were hard to identify, and most of them occurred in low concentrations. Their total content measured 28.65 mg/g (expressed as isorhamnetin 3-*O*-Glc(1→2)-Gal equivalent).

**Figure 1:**
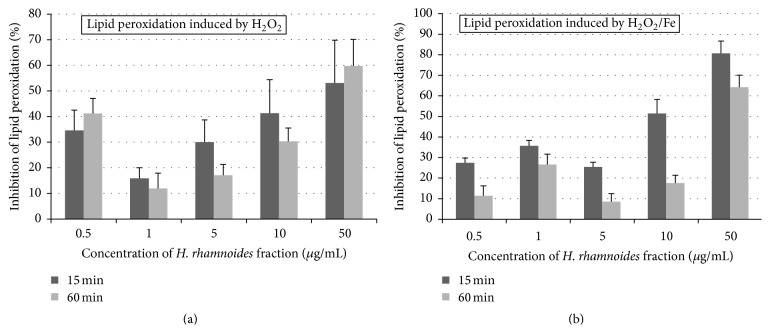
Effects of the phenolic fraction from fruits of* H. rhamnoides* (0.5–50 *µ*g/mL; 15 and 60 min) on plasma lipid peroxidation induced by H_2_O_2_ (a) and plasma lipid peroxidation induced by H_2_O_2_/Fe (b). Data represent means ± standard error (SE) of 4-5. The effect of five different concentrations of the tested fraction (0.5, 1, 5, 10, and 50 *μ*g/mL) was statistically significant according to ANOVA *I* test, *p* < 0.05 for concentrations 0.5, 5, 10, and 50 *μ*g/mL (for 15 and 60 min) (a); *p* > 0.05 for concentration 1 *μ*g/mL (for 15 and 60 min) (a); *p* < 0.05 for concentrations 0.5, 1, and 5 *μ*g/mL (for 15 min) (b); *p* < 0.02 for concentrations 10 and 50 *μ*g/mL (for 15 min) (b); *p* < 0.02 for concentration 50 *μ*g/mL (for 60 min) (b); *p* > 0.05 for concentrations 0.5, 1, 5, and 10 *μ*g/mL (for 60 min) (b).

**Figure 2:**
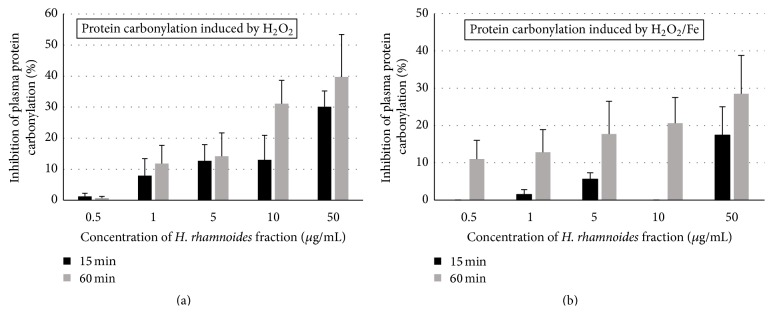
Effects of the phenolic fraction from fruits of* H. rhamnoides* (0.5–50 *µ*g/mL; 15 and 60 min) on plasma protein carbonylation induced by H_2_O_2_ (a) and plasma protein carbonylation induced by H_2_O_2_/Fe (b). Data represent means ± standard error (SE) of 4. The effect of five different concentrations of the tested fraction (0.5, 1, 5, 10, and 50 *μ*g/mL) was statistically significant according to ANOVA *I* test, *p* < 0.05 for concentration 50 *μ*g/mL (for 15 min) (a); *p* < 0.05 for concentrations 10 and 50 *μ*g/mL (for 60 min) (a); *p* > 0.05 for concentrations 0.5, 1, 5, and 10 *μ*g/mL (for 15 min) (a); *p* > 0.05 for concentrations 0.5, 1, and 5 *μ*g/mL (for 60 min) (a); *p* < 0.05 for concentration 50 *μ*g/mL (for 15 min) (b); *p* < 0.05 for concentrations 5, 10, and 50 *μ*g/mL (for 60 min) (b); *p* > 0.05 for concentrations 0.5, 1, 5, and 10 *μ*g/mL (for 15 min) (b); *p* > 0.05 for concentrations 0.5 and 1 *μ*g/mL (for 60 min) (b).

The antioxidative activities of the phenolic fraction from* H. rhamnoides* fruits (at a dose range 0.5–50 *μ*g/mL; incubation time: 15 and 60 min) were studied* in vitro*. As demonstrated in Figures 1 and 2, the tested fraction inhibited plasma lipid peroxidation and protein carbonylation stimulated by H_2_O_2_ or H_2_O_2_/Fe. The inhibition of lipid peroxidation in plasma reached about 60% when the highest concentration (50 *μ*g/mL) of the fraction and the longest incubation time (60 min) were applied (Figures 1(a) and 1(b)).

**Figure 3:**
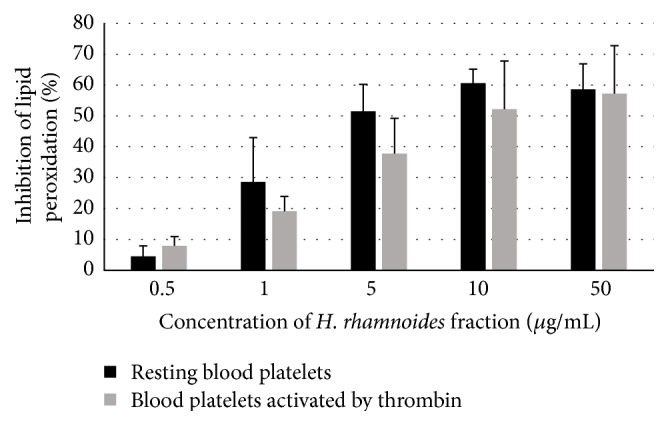
Effects of the phenolic fraction from fruits of* H. rhamnoides* (0.5–50 *µ*g/mL; 15 min) on lipid peroxidation in resting blood platelets and blood platelets activated by thrombin. Data represent means of 3-4 donors ± standard error (SE). The effect of five different concentrations of the tested fraction (0.5, 1, 5, 10, and 50 *μ*g/mL) was statistically significant according to ANOVA *I* test, *p* < 0.05 for concentrations 1, 5, 10, and 50 *μ*g/mL; *p* > 0.05 for concentration 0.5 *μ*g/mL.

Another set of experiments focused on the determination of TBARS levels as a measure of nonenzymatic lipid peroxidation in resting blood platelets and enzymatical peroxidation of arachidonic acid in blood platelets stimulated by thrombin. After a 15 min preincubation of platelets with the* H. rhamnoides* phenolic fraction, the amount of TBARS in resting platelets and thrombin-activated platelets diminished. The fraction's activity was concentration-dependent (Figure 3). At the highest concentration of the tested fraction (50 *µ*g/mL), production of TBARS in resting and activated platelets was reduced by about 60% (Figure 3).

**Figure 4:**
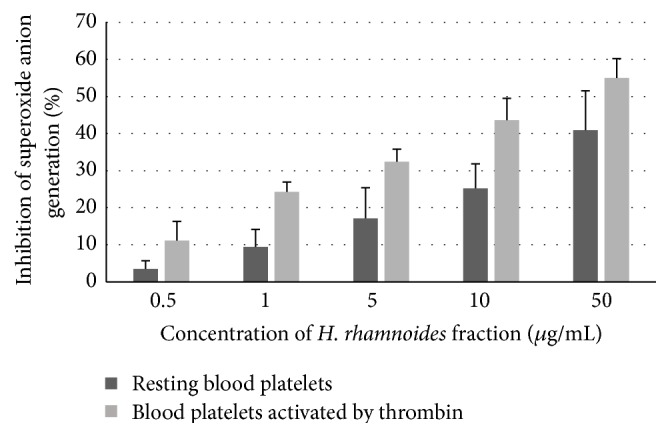
Effects of the phenolic fraction from fruits of* H. rhamnoides* (0.5–50 *µ*g/mL; 15 min) on O_2_^−∙^ generation in resting blood platelets and blood platelets activated by thrombin. Data represent means of 4–6 donors ± standard error (SE). The effect of five different concentrations of the tested fraction (0.5, 1, 5, 10, and 50 *μ*g/mL) was statistically significant according to ANOVA *I* test, *p* < 0.05 for concentrations 5, 10, and 50 *μ*g/mL (resting platelets); *p* < 0.05 for concentrations 1, 5, 10, and 50 *μ*g/mL (platelets activated by thrombin); *p* > 0.05 for concentration 0.5 *μ*g/mL (resting platelets and platelets activated by thrombin); *p* > 0.05 for concentration 1 *μ*g/mL (resting platelets).

Analysis of the effect of* H. rhamnoides* phenolic extract (at concentrations between 0.5 and 50 *µ*g/mL) on the reduction of O_2_^−∙^) generation in resting blood platelets and platelets activated by thrombin is shown in Figure 4. All investigated concentrations of the phenolic fraction decreased O_2_^−∙^ production. The strongest inhibition was observed in platelets treated with the highest dose (50 *µ*g/mL) of the fraction, reaching about 40% for resting platelets and about 55% for platelets activated by thrombin (Figure 4).

**Figure 5:**
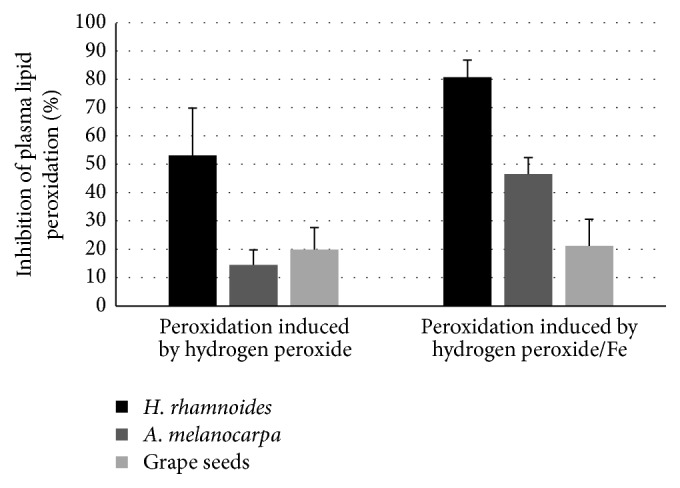
Effects of the phenolic fraction from fruits of* H. rhamnoides *(50 *µ*g/mL; 15 min),* A. melanocarpa* extract (50 *µ*g/mL, 15 min), and grape seed extract (50 *µ*g/mL, 15 min) on plasma lipid peroxidation induced by H_2_O_2_ or H_2_O_2_/Fe. The results represent 3–8 independent experiments and are expressed as means ± SE. The effects were significant according to ANOVA *I* test, for peroxidation induced by H_2_O_2_:* H. rhamnoides *fraction-treated plasma* versus A. melanocarpa* extract-treated plasma (*p* < 0.02);* H. rhamnoides *fraction-treated plasma* versus* grape seed extract-treated plasma (*p* < 0.05); for peroxidation induced by H_2_O_2_/Fe:* H. rhamnoides *fraction-treated plasma* versus A. melanocarpa* extract-treated plasma (*p* < 0.01);* H. rhamnoides *fraction-treated plasma* versus* grape seed extract-treated plasma (*p* < 0.01).

**Figure 6 fig6:**
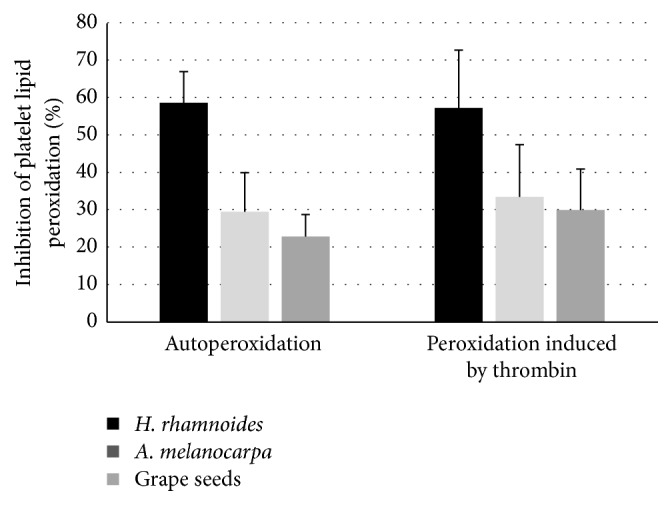
Effects of the phenolic fraction from fruits of* H. rhamnoides *(50 *µ*g/mL; 15 min),* A. melanocarpa* extract (50 *µ*g/mL, 15 min), and grape seed extract (50 *µ*g/mL, 15 min) on autoperoxidation of blood platelets and platelet lipid peroxidation induced by thrombin. The results represent 3-4 independent experiments and are expressed as means ± SE. The effects were significant according to ANOVA *I* test, for autoperoxidation:* H. rhamnoides *fraction-treated platelets* versus A. melanocarpa* extract-treated platelets (*p* < 0.01);* H. rhamnoides *fraction-treated platelets* versus* grape seed extract-treated platelets (*p* < 0.01); for peroxidation induced by thrombin:* H. rhamnoides *fraction-treated platelets* versus A. melanocarpa* extract-treated platelets (*p* < 0.05);* H. rhamnoides *fraction-treated platelets* versus* grape seed extract-treated platelets (*p* < 0.05).

**Figure 7:**
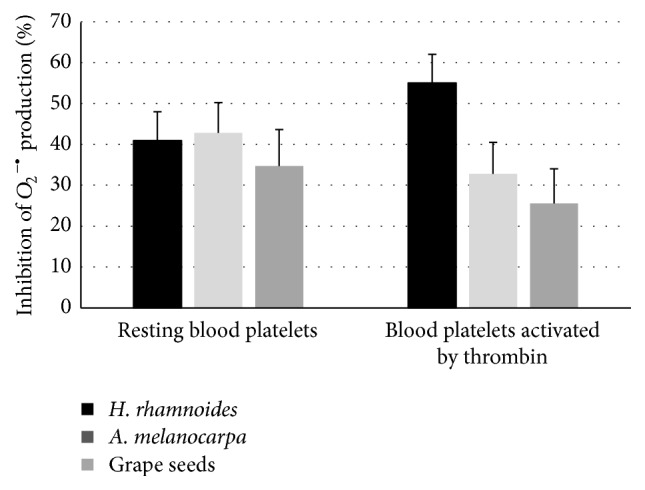
Effects of the phenolic fraction from fruits of* H. rhamnoides *(50 *µ*g/mL; 15 min),* A. melanocarpa* extract (50 *µ*g/mL, 15 min), and grape seed extract (50 *µ*g/mL, 15 min) on O_2_^−∙^ generation in resting blood platelets and blood platelets activated by thrombin. The results represent 3–6 independent experiments and are expressed as means ± SE. The effects were significant according to ANOVA *I* test, for resting platelets:* H. rhamnoides *fraction-treated platelets* versus A. melanocarpa* extract-treated platelets (*p* > 0.05);* H. rhamnoides *fraction-treated platelets* versus* grape seed extract-treated platelets (*p* > 0.05); for peroxidation induced by thrombin:* H. rhamnoides *fraction-treated platelets* versus A. melanocarpa* extract-treated platelets (*p* < 0.05);* H. rhamnoides *fraction-treated platelets* versus* grape seed extract-treated platelets (*p* < 0.05).

In comparative experiments, the phenolic fraction of sea buckthorn fruits (at the highest tested concentration, 50 *µ*g/mL) turned out to be more effective than 50 *µ*g/mL* Aronia* extract or 50 *µ*g/mL grape seed extract (Figures 5 [Fig fig6]–7).

## 4. Discussion

Phenolic compounds are plant secondary metabolites with antioxidant properties. They play an important role in adsorbing and neutralizing reactive oxygen species. Moreover, phenols may act as chelators for the transition of metal ions Fe^2+^, Fe^3+^, and Cu^2+^ that are involved in the conversion of H_2_O_2_ into OH^•^ and stimulation of lipid peroxidation [24]. Prooxidative or antioxidative activities of the phenolic fraction from fruits of* H. rhamnoides* and their biological significance remain unclear. Only few human experiments were found, which examine the effects of* H. rhamnoides* on the oxidative stress associated with cardiovascular diseases [25]. The used* H. rhamnoides* fraction from fruits was tested at a dose range of 0.5–50 *µ*g/mL, which corresponds to the physiological range of phenolic compounds in human plasma. Plasma and blood platelets were used in our* in vitro* study because they are vital components of hemostasis; moreover, oxidative stress in plasma and blood platelets may promote the development of cardiovascular diseases. In our experiments, addition of H_2_O_2_ or H_2_O_2_/Fe to human plasma resulted in a significant increase in the level of oxidative stress (measured by TBARS or the level of carbonyl groups). Our findings demonstrate for the first time the antioxidative properties of the phenolic fraction from* H. rhamnoides *fruits. The tested fraction significantly inhibited plasma lipid peroxidation induced by H_2_O_2_ or H_2_O_2_/Fe.

Protein carbonyl formation is a relatively stable biomarker of oxidative stress, as a result of amino acid modifications (i.e., Lys, Arg, Cys, Thr, or Pro). Protein carbonylation is the nonenzymatic addition of aldehydes or ketones to specific amino acid residues. In our experiments, the phenolic fraction from fruits of* H. rhamnoides* decreased the concentration of carbonyl groups in plasma proteins treated with H_2_O_2_ or H_2_O_2_/Fe. Our findings show for the first time that the phenolic fraction of* H. rhamnoides* fruits has inhibitory action not only on human plasma lipid peroxidation, but also on carbonylation of human plasma proteins. Upadhyay et al. [9] observed the said antioxidant effect of* H. rhamnoides* leaves. Aqueous and hypoalcoholic extracts of* H. rhamnoides* had cytoprotective activity against hydrogen peroxide and hypoxanthine-xanthine oxidase-stimulated damage to BHK-21 cell line. Maheshwari et al. [26] demonstrated that the phenolic-rich fraction of* H. rhamnoides* leaves has potent antioxidant activity, prevents oxidative damage to lipids and proteins, and affords significant protection against CCl_4_-stimulated oxidative liver damage in Sprague Dawley rats. Based on the results of Khan et al. [27], it can be shown that* H. rhamnoides* leaf extract ameliorates gamma radiation-mediated DNA damage and hepatic alterations.

Another set of our experiments showed that the* H. rhamnoides* phenolic fraction could effectively reduce lipid peroxidation in blood platelets. After 15 min preincubation of platelets with the tested fraction, the amounts of TBARS in resting and thrombin-stimulated platelets were reduced. The results suggest that the tested extract may also play a role in modulating blood platelet activation by interfering with the metabolism of arachidonic acid, in which cyclooxygenase or lipoxygenase takes part. In addition, blood platelets themselves can produce several ROS, including the superoxide anion, hydrogen peroxide, and hydroxyl radical [22, 28]. It has been demonstrated that ROS participate in signal transmission. Changes in blood platelet responsiveness to a strong physiological agonist, thrombin, were observed in the present* in vitro* study, when platelets were preincubated with the* H. rhamnoides *fraction. Because cyclooxygenase is involved in ROS generation in platelets, it seems possible that the tested fraction may modulate ROS production by interfering with the metabolism of arachidonic acid, in which this enzyme takes part. However, results obtained by Eccleston et al. [11] showed that there were no significant changes in blood platelet aggregation between treatment groups (twenty healthy male volunteers were given either a placebo or* H. rhamnoides *juice for 8 weeks). On the other hand,* H. rhamnoides *seed oil has antiatherogenic and cardioprotective activity [5, 29]. Moreover, an antihypertensive effect of flavones extracted from seed residues of* H. rhamnoides* in sucrose-fed rats was observed by Pang et al. [1].

Additionally, we studied the effect of the tested fraction on O_2_^−∙^ production in resting blood platelets. The fraction exhibited antioxidant activity not only in blood platelets activated by thrombin, but also in resting platelets. Our results also demonstrated that the phenolic fraction from* H. rhamnoides* fruits had stronger antioxidant activity than extracts from* Aronia* berries and grape seeds. It may be supposed that differences in their chemical profiles (the total concentration of phenolics for* Aronia* berry extract, 309.6 mg/g of extract; the total concentration of phenolics for grape seed extract, 500 mg/g of extract; the total concentration of phenolics for* H. rhamnoides *fruits fraction, 242.69 mg/g of extract) may explain the stronger action of the* H. rhamnoides *fraction. The major phenolic compounds of* H. rhamnoides* fraction are the flavonols. Flavonol glycosides, isorhamnetin 3-*O*-hexoside-deoxyhexoside and isorhamnetin 3-*O*-hexoside, were present in the highest concentrations. Teleszko et al. [30] also identified in* H. rhamnoides* fruits flavonols as the major group of phenolic compounds. We suppose that these phenolic compounds may act as main antioxidants in* H. rhamnoides* fraction. It is important to note that other tested extracts have not these compounds. Therefore, the effects of various phenolic compounds, that is, isorhamnetin 3-*O*-hexoside-deoxyhexoside and isorhamnetin 3-*O*-hexoside, which may be responsible for antioxidative and antiplatelet activity, remain to be investigated. Moreover, results of Luo et al. [31] indicate that isorhamnetin inhibited atherosclerotic plaque development by phosphatidylinositol 3-kinase/protein kinase B activation and heme oxygenase induction.

Our present results show that* H. rhamnoides* fraction, like* Aronia* berries extract and grape seeds extract, displays a multiple effect that is on blood platelets.* H. rhamnoides* fraction may modulate the signal transduction in different, sometimes opposite, pathways. Firstly, it causes changes in reactive oxygen species level in platelets. Secondly, it modulates the arachidonic acid metabolism (probably by the inhibition of cyclooxygenase activity). However, it may also change the expression of receptors or activity of various enzymes involved in platelet activation.

The fraction of* H. rhamnoides* fruits in comparison to a well-known* Aronia* berries may be a good source of active substances for pharmacological or cosmetic applications. From an economic point of view, it is true that* H. rhamnoides* fruits purchase price is about four times higher than* Aronia* berries price, but the yield per hectare of* H. rhamnoides* fruits is four times larger at the same time. Our preliminary studies show that the* H. rhamnoides* leaves contain qualitatively the same compounds as their fruits. As fruits are harvested together with leaves and branches, on which they grow, there is a possibility of obtaining additionally phenolic compounds from leaves as a production waste.

Our study demonstrates that* H. rhamnoides *fruits could be used as a natural source of antioxidants and compounds with antiplatelet activity to prevent and/or cure disorders associated with oxidative stress and changes in platelet activation. Moreover, it gives hope for the development of new diet supplements, and so experiments with the fruit of* H. rhamnoides *should be continued. We plan to identify individual or groups of phenolic compounds, which are responsible for the antioxidant and antiplatelet properties of* H. rhamnoides* fraction.

## Figures and Tables

**Table 1:** Content of flavonol glycosides in the phenolic fraction of sea buckthorn fruits.

	*t*_*R*_	[M−H]^−^	mg/g ± SD
Quercetin 3-*O*-Hex-Hex-7-*O*-dHex^*∗*^	6.62	771	2.03 ± 0.02
Quercetin 3-*O*-Hex-Hex-7-*O*-dHex^*∗*^	6.75	771	8.13 ± 0.05
Kaempferol 3-*O*-Hex-Hex-dHex	7.07	755	0.99 ± 0.19
Kaempferol 3-*O*-Hex-Hex-dHex	7.23	755	9.45 ± 0.07
Isorhamnetin 3-*O*-Hex-Hex-dHex	7.53	785	3.86 ± 0.00
Isorhamnetin 3-*O*-Hex-Hex-dHex	7.59	785	15.36 ± 0.27
Quercetin 3-*O*-Hex-7-O-dHex^*∗*^	8.14	609	3.38 ± 0.04
Quercetin 3-*O*-Hex-dHex-dHex	8.20	755	5.62 ± 0.04
Quercetin 3-*O*-Hex-dHex	9.17	609	10.00 ± 0.10
Isorhamnetin 3-*O*-Hex-7-*O*-dHex^*∗*^	9.26	623	33.57 ± 0.14
Quercetin 3-*O*-Hex	9.46	463	25.32 ± 0.15
Isorhamnetin 3-*O*-Hex-dHex	10.32	623	3.75 ± 0.13
Isorhamnetin 3-*O*-Hex-dHex	10.50	623	44.00 ± 0.35
Isorhamnetin 3-*O*-Hex	10.85	477	44.16 ± 0.08
Quercetin derivative	12.00	709	0.64 ± 0.01
Isorhamnetin derivative	12.95	723	1.94 ± 0.13
Isorhamnetin derivative	13.76	723	1.84 ± 0.02
Other flavonol glycosides			28.65 ± 0.94

^*∗*^Position of sugar groups determined on the basis of literature data; Hex: a hexose; dHex: a deoxyhexose.
